# Supplementary figures and images for: Adenine methylation is very scarce in the Drosophila genome and not erased by the ten-eleven translocation dioxygenase
Source: eLife. 2023 Dec 21;12:RP91655. doi: 10.7554/eLife.91655 (PMC10735219; doi:10.7554/eLife.91655)

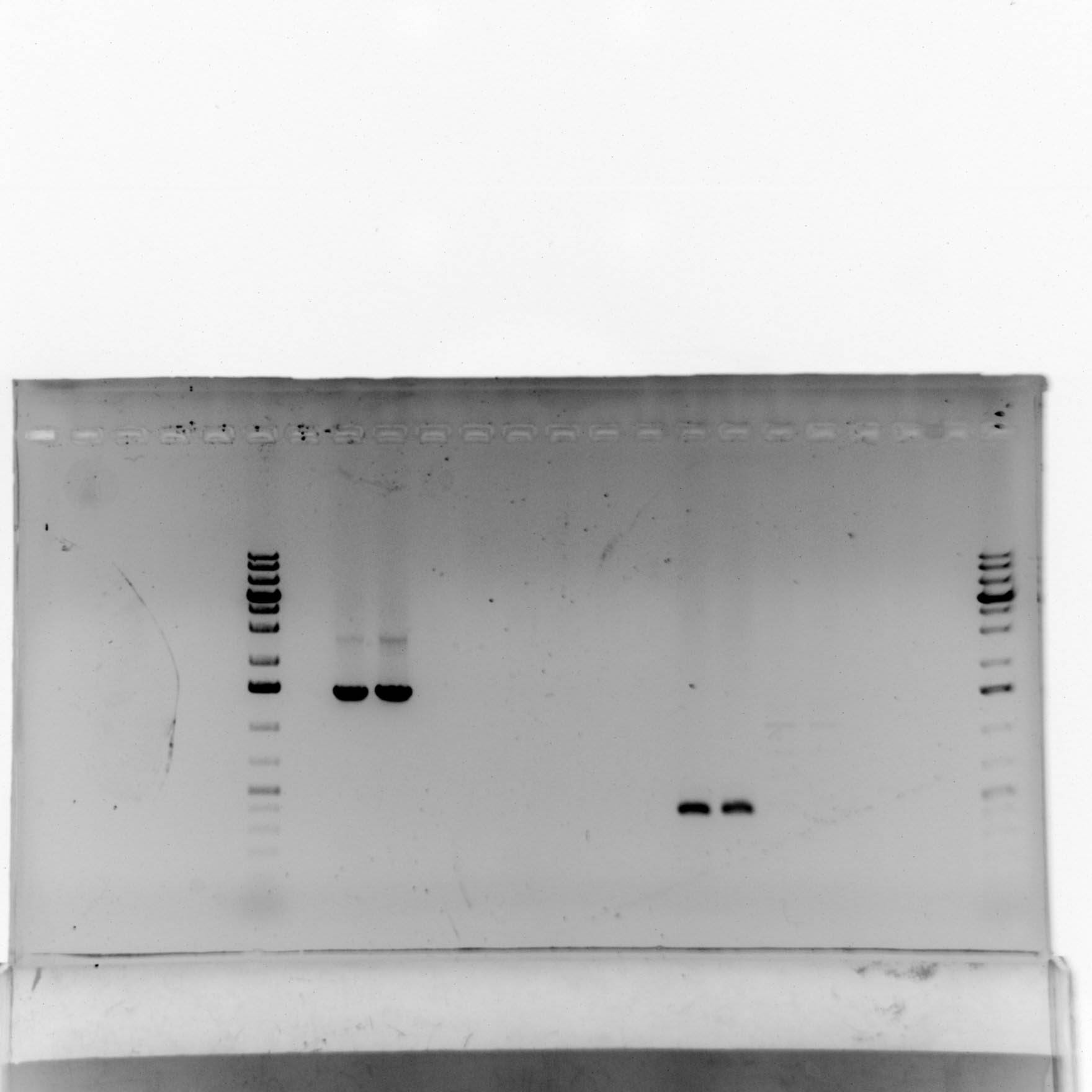

Supplement: Figure 1—source data 1. [file elife-91655-fig1-data1.zip › Fig 1 source data 1.jpg]

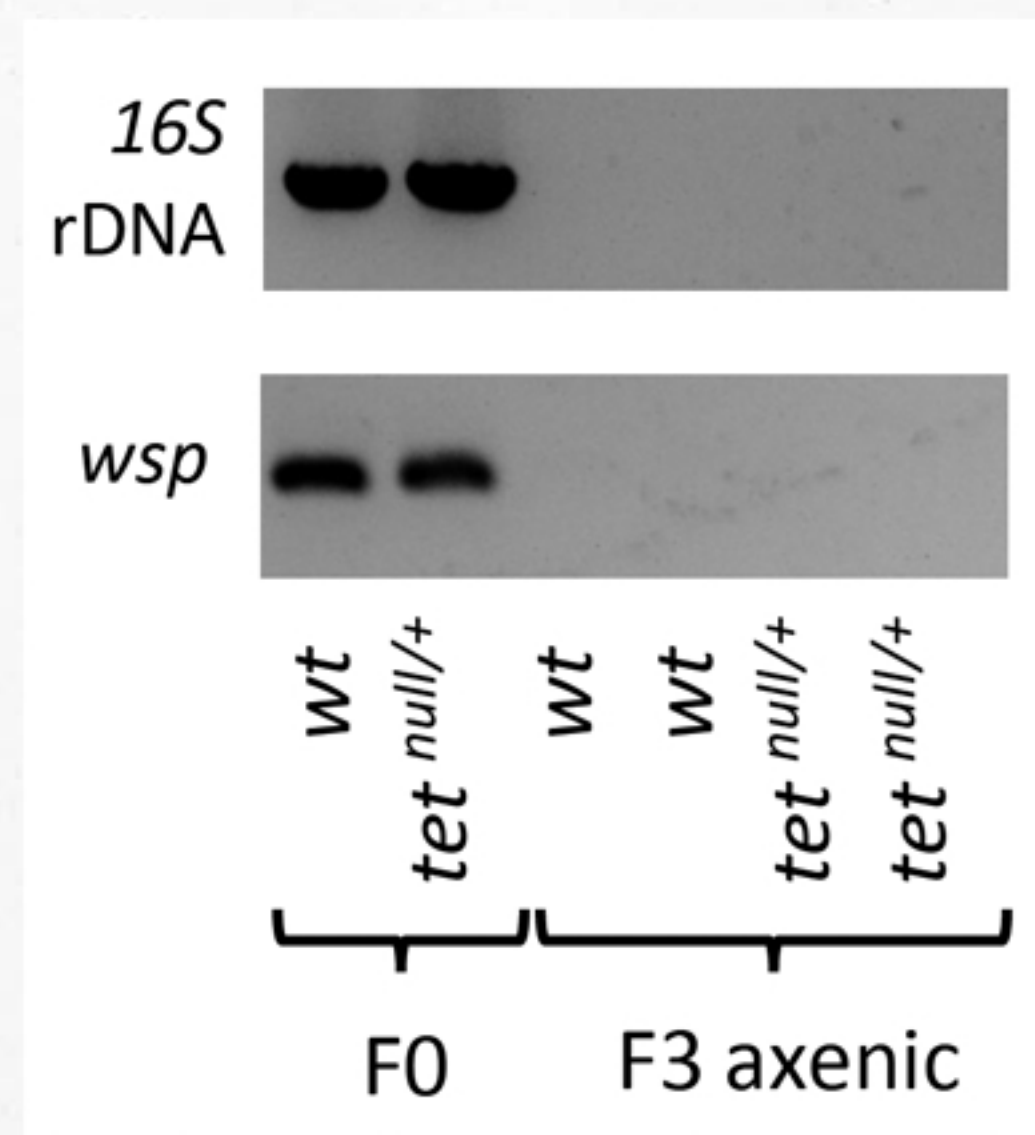

Lon

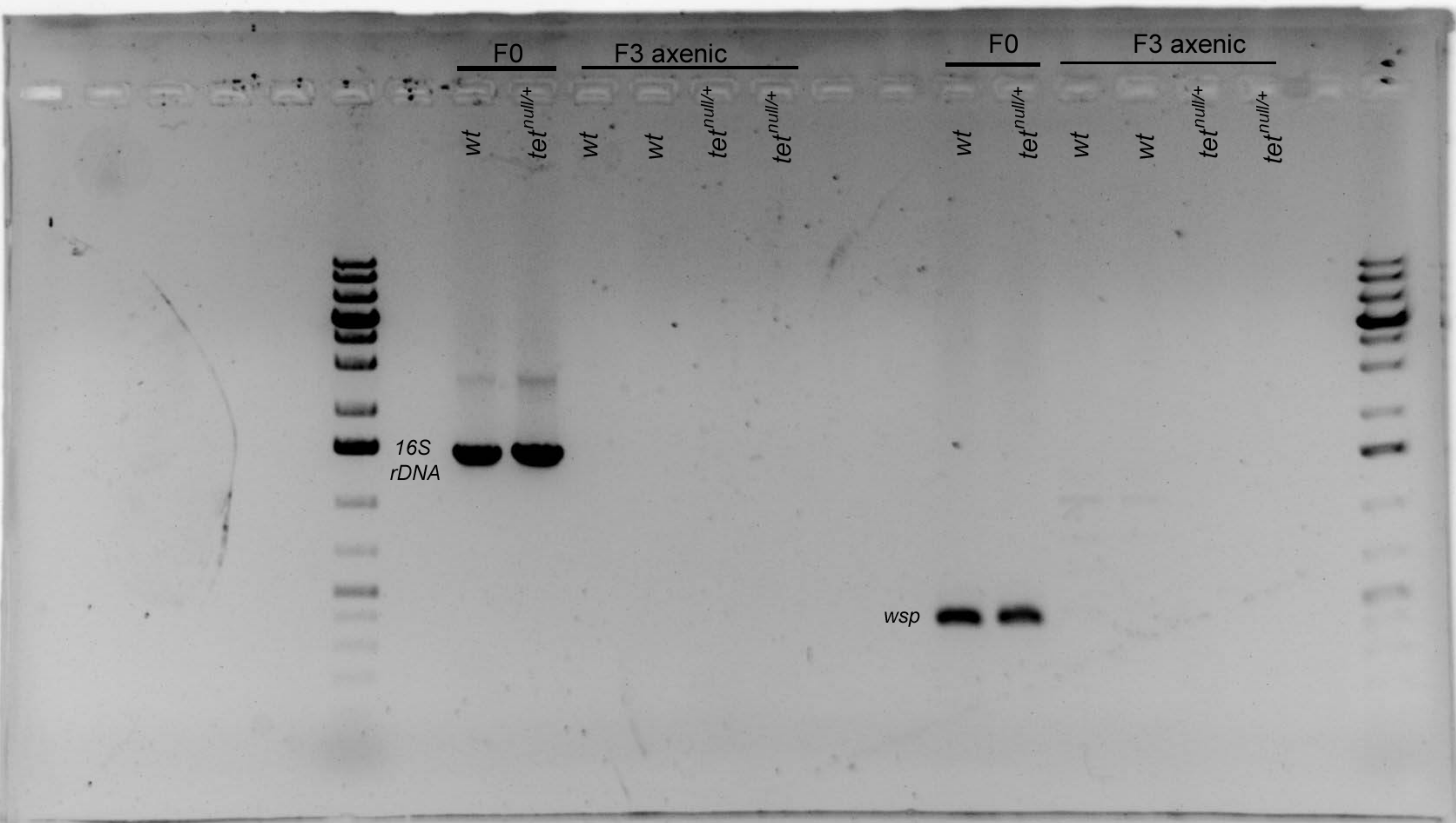

Supplement: Figure 1—source data 2. [file elife-91655-fig1-data2.zip › Fig 1 source data 2.pdf]

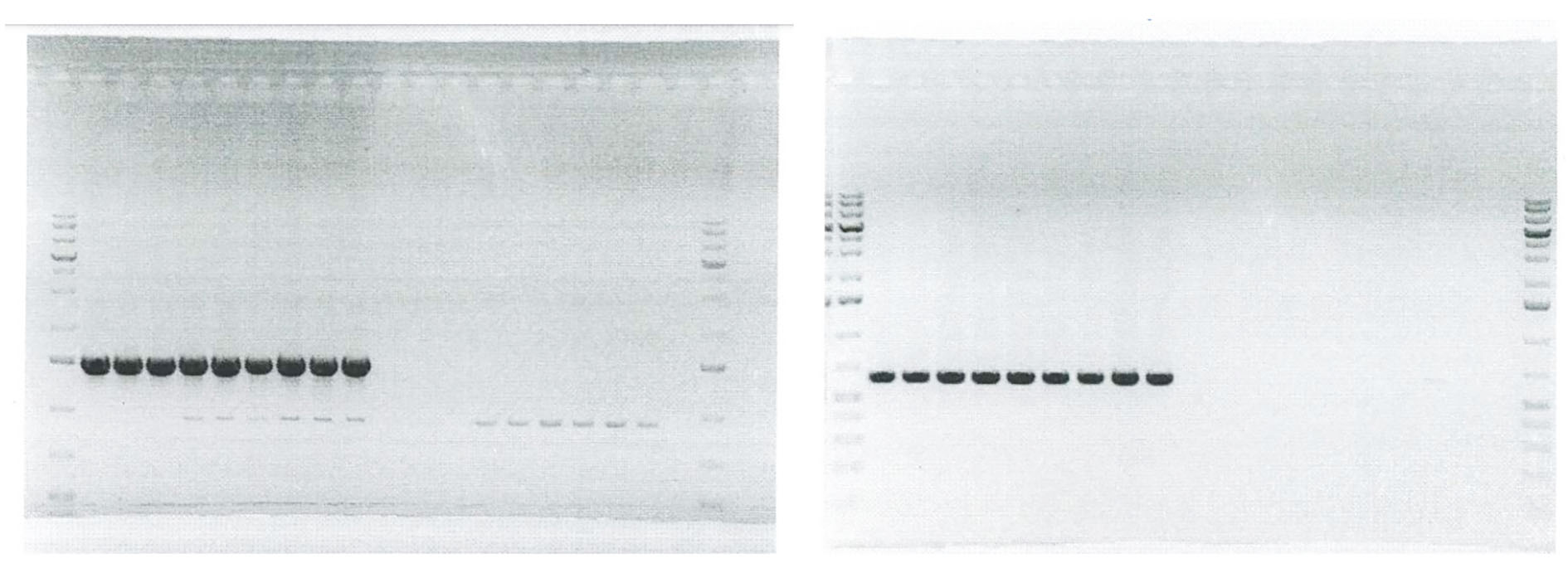

Supplement: Figure 1—figure supplement 1—source data 1. [file elife-91655-fig1-figsupp1-data1.zip › Fig 1-figsup 1 source data 1.jpg]

a

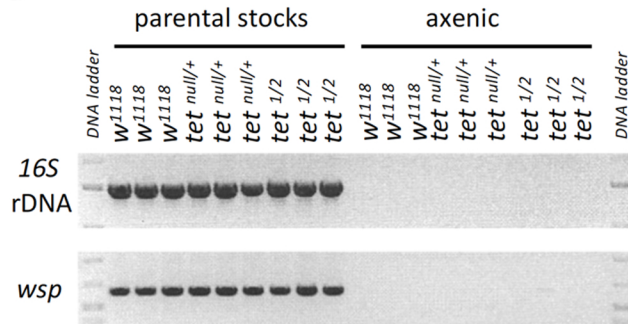

16S rDNA

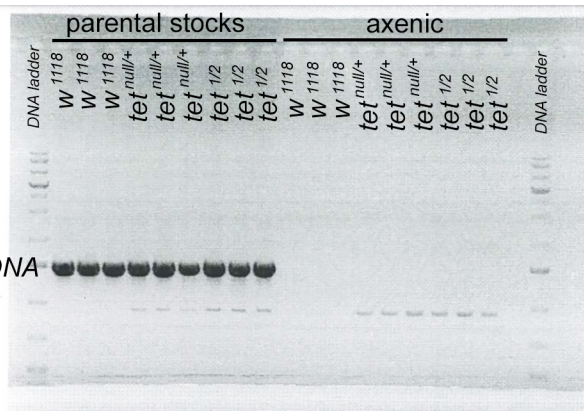*wsp*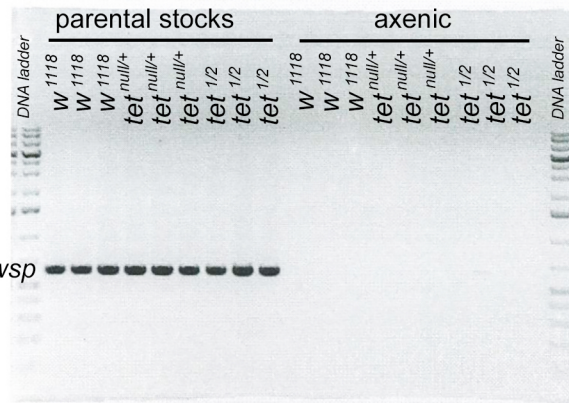

Supplement: Figure 1—figure supplement 1—source data 2. [file elife-91655-fig1-figsupp1-data2.zip › Fig 1-figsup 1 source data 2.pdf]
